# *In Silico* Identification and *in Vitro* Activity of Novel Natural Inhibitors of *Trypanosoma brucei* Glyceraldehyde-3-phosphate-dehydrogenase †

**DOI:** 10.3390/molecules200916154

**Published:** 2015-09-03

**Authors:** Fabian C. Herrmann, Mairin Lenz, Joachim Jose, Marcel Kaiser, Reto Brun, Thomas J. Schmidt

**Affiliations:** 1 Institut für Pharmazeutische Biologie und Phytochemie (IPBP), University of Münster, PharmaCampus, Correnstraße 48, Münster D-48149, Germany; E-Mails: f_herr01@uni-muenster.de (F.C.H.); m_lenz01@uni-muenster.de (M.L.); 2 Institut für Pharmazeutische und Medizinische Chemie, University of Münster, PharmaCampus, Correnstraße 48, Münster D-48149, Germany; E-Mail: joachim.jose@uni-muenster.de; 3 Swiss Tropical and Public Health Institute (Swiss TPH), Socinstraße 57, Basel CH-4002, Switzerland; E-Mails: Marcel.Kaiser@unibas.ch (M.K.); Reto.Brun@unibas.ch (R.B.); 4 University of Basel, Petersplatz 1, Basel CH-4003, Switzerland

**Keywords:** *Trypanosoma brucei*, human African trypanosomiasis, glyceraldehyde-3-phosphate dehydrogenase inhibitor, natural product, *in silico* screening, *in vitro* antitrypanosomal activity

## Abstract

As part of our ongoing efforts to identify natural products with activity against pathogens causing neglected tropical diseases, we are currently performing an extensive screening of natural product (NP) databases against a multitude of protozoan parasite proteins. Within this project, we screened a database of NPs from a commercial supplier, AnalytiCon Discovery (Potsdam, Germany), against *Trypanosoma brucei* glyceraldehyde-3-phosphate dehydrogenase (*Tb*GAPDH), a glycolytic enzyme whose inhibition deprives the parasite of energy supply. NPs acting as potential inhibitors of the mentioned enzyme were identified using a pharmacophore-based virtual screening and subsequent docking of the identified hits into the active site of interest. In a set of 700 structures chosen for the screening, 13 (1.9%) were predicted to possess significant affinity towards the enzyme and were therefore tested in an *in vitro* enzyme assay using recombinant *Tb*GAPDH. Nine of these *in silico* hits (69%) showed significant inhibitory activity at 50 µM, of which two geranylated benzophenone derivatives proved to be particularly active with IC_50_ values below 10 µM. These compounds also showed moderate *in vitro* activity against *T. brucei rhodesiense* and may thus represent interesting starting points for further optimization.

## 1. Introduction

*Trypanosoma brucei*, the pathogen responsible for human African trypanosomiasis (HAT, or “sleeping sickness”), is widely spread in Sub-Saharan Africa and endangering about 70 million people with a possible infection vectored by the bite of the Tsetse-fly (*Glossina* spp., family Glossinidae), causing an estimated incidence of 20,000 acute cases per year [1].

The rate of mortality of HAT is 100%, when left untreated. Available chemotherapeutics, however, suffer from lack of efficacy, limited availability/applicability, poor safety profiles and emerging resistances. Additionally, their mechanisms of action are mostly not known. Because mainly poor and rural populations are affected by HAT, research and development efforts for new therapeutics do not promise economic success. All of these facts together are the reasons that HAT is included among the “Neglected Tropical Diseases” (NTDs) by the WHO and underline the urgent need for the development of new antitrypanosomal agents for chemotherapy.

Natural products offer a vast diversity of chemical structures that often show a high potential as new scaffolds or leads for new drugs [2]. This has also been pointed out in particular with respect to protozoan infectious diseases [3,4]. Besides the more traditional approach of screening natural products in phenotypic assays directly against the parasites (e.g., [5,6,7,8,9]), our group also applies more rational computer-based methods to the search for new natural compounds with anti-protozoal activity, e.g., [10,11].

One strategy to fight trypanosomatid parasites is to target their peculiar energy metabolism. Members of the family Trypanosomatidae depend entirely on glycolysis for the acquisition of nucleoside triphosphates, *i.e.*, energy supply. The relative importance of this pathway is believed to have resulted in a particular, highly-specialized compartmentation within the parasite cell. The most prominent trypanosomatid peculiarity is a peroxisome-like organelle called the glycosome, hosting most of the glycolytic enzymes and allowing the pathogen to rapidly adapt its metabolism to changing the host species during its life cycle [12,13]. Among some other glycolytic enzymes, glyceraldehyde-3-phosphate dehydrogenase (GAPDH) has been suggested as a potential drug target [14], and this role has also been validated genetically by RNAi experiments [15]. This is emphasized also by the fact that the inhibition of erythrocytic GAPDH (up to 95%) in mammals does not cause a severe lack of ATP [16]. Inhibitors of trypanosomatid GAPDH may therefore represent promising lead structures for the design of innovative trypanocidal agents [14,15,17]. Up to now, only relatively few inhibitors of trypanosomatid GAPDH are known. Among these are several natural products (for an overview, see [3]), comprising a diterpene [18] and a few triterpenes [19], some indole alkaloids [20], some flavonoids [21], as well as coumarin derivatives [22].

As part of our ongoing efforts to identify natural products with activity against pathogens causing neglected tropical diseases using structure-based methods [23], we are currently performing an extensive screening of natural product (NP) databases against a multitude of protozoan parasite proteins. In the present communication, we report on the identification of natural products (NPs) with inhibitory activity towards the GAPDH of *Trypanosoma brucei* (*Tb*GAPDH), discovered by means of an *in silico* screening of a virtual NP library consisting of 700 compounds. The predictions resulting from pharmacophore-based virtual screening followed by molecular docking were confirmed by a spectrophotometric inhibition assay in which the best hits of the *in silico* study were tested against recombinant *Tb*GAPDH. These hits were also found moderately active *in vitro* against *T. brucei rhodesiense* and may therefore represent interesting starting points for further development.

## 2. Results and Discussion

### 2.1. In Silico Prediction of Potential TbGAPDH Inhibitors

A virtual database of commercially available natural products (MEGx library) supplied by AnalytiCon Discovery GmbH (Potsdam, Germany) consisting of 4803 natural compounds served as the basis for the identification of new inhibitors of *Tb*GAPDH, employing a virtual screening (VS) approach by means of the software MOE [24]. Compounds not likely to possess drug-like properties according to Lipiski’s rule were removed from the collection. In a large commercial database of natural products as the one used here, problems may arise from compounds with unclear assignment of chirality at particular stereocenters or in cases where only relative stereochemistry is known. Moreover, achiral compounds are more easily accessible to synthesis in the case of hits that require larger amounts of material for further development. We therefore also decided to remove chiral compounds from the database, which finally resulted in a subset of 700 NPs, which, after full geometry optimization, constituted the final collection of NPs for the virtual screening protocol. Three GAPDH structures from different trypanosomatid pathogens responsible for human infections (*Trypanosoma brucei*, *Trypanosoma cruzi* and *Leishmania mexicana*; PDB-IDs are given in the Experimental Section) were retrieved from the Protein Data Bank [25]. The protein structures, after correction and energy minimization, were used to construct pharmacophore queries and, subsequently, to perform docking studies. In all three protein structures, multiple highly-conserved catalytic sites were present, each containing one co-crystallized molecule of the cosubstrate NAD^+^. Even though it might appear somewhat problematic to search for inhibitors addressing the cosubstrate binding site, which is defined by a similar overall fold in all dehydrogenase enzymes [26], there appear to be various differences in the detailed amino acid composition between the various related enzymes of different organisms. Differences in the binding site between trypanosomatid and human enzyme have been described and already exploited previously for the design of selective inhibitors of trypanosomal GAPDH [17]. We therefore decided to include, besides the substrate binding site (G-3-P site), the NAD^+^-binding site in our virtual screening/docking. The experimentally-determined natural cosubstrate structure thus served as a template for the design of pharmacophore queries to address the NAD^+^ binding site in the VS. Due to the fact that none of the protein structures contained a co-crystallized molecule of the substrate G-3-P, the substrate binding site was not accessible by this strategy. In order to include this most important structural element in the VS, a pharmacophore query for the G-3-P site of *Tb*GAPDH was compiled by means of surface analysis (*i.e*., using the “electrostatic map” feature implemented in MOE; a representation of the G-3-P pharmacophore query is given in Figure S3, Supplementary Information). Altogether, four pharmacophore queries were created (one for the closely-related, but slightly differing NAD^+^ site of each parasite species’ enzyme, as well as one for the G-3-P site of *Tb*GAPDH), allowing the VS of the mentioned database. Each pharmacophore was used to perform a separate VS of the NP database.

Each set of hits was then docked (rigid docking) into the respective enzyme/binding site on which its pharmacophore query was based, resulting in a ranking based on the calculated docking score (S-score, in kcal/mol). The ten compounds with the lowest S-score at each of the binding sites were combined into a collection of “consensus hits”, which was then subject to a more refined docking analysis (induced fit approach, *i.e*., both the binding site and the ligand were treated as flexible structures). Based on the *S-*scores of this final docking step with *Tb*GAPDH, the most promising hits were selected for *in vitro* testing.

**Figure 1:**
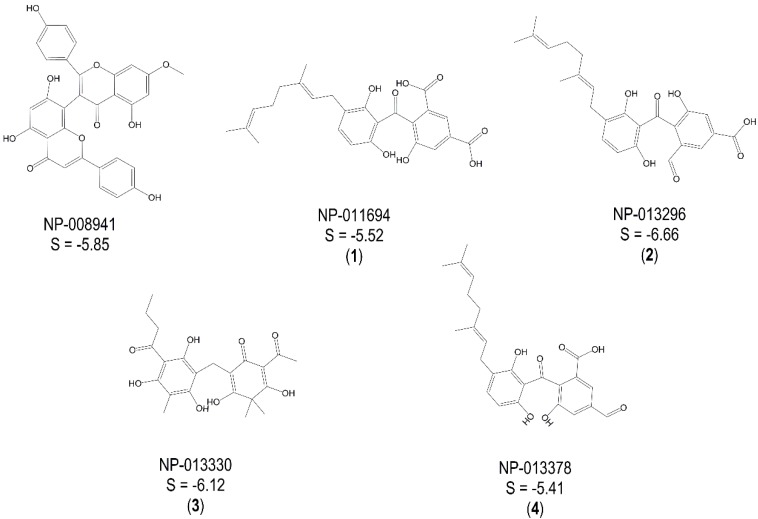
Best hits from the virtual screening based on the G-3-P pharmacophore; S-scores of the docking experiment (induced-fit approach) are given in kcal/mol; a “self-docking” computation of the cosubstrate d-glyceraldehyde-3-phosphate was performed accordingly (S_G-3-P_ = −3.81 kcal/mol). NP, natural product.

**Figure 2:**
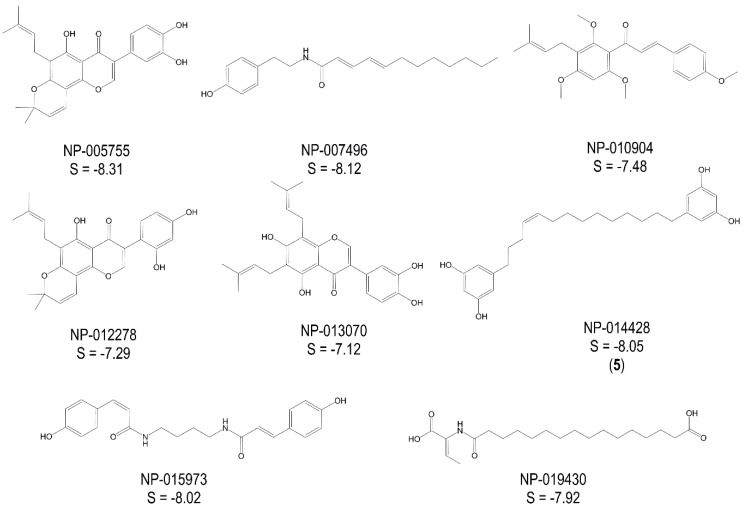
Best hits from the virtual screening based on the NAD^+^ pharmacophores; S-scores of the docking experiments (induced-fit approach) are given in kcal/mol; a “self-docking” computation of the cosubstrate was performed accordingly (S_NAD+_ = −9.10 kcal/mol).

Figure 1 shows the best hits obtained by the G-3-P pharmacophore and the corresponding calculated docking scores (S in kcal/mol) after induced fit docking into the G-3-P-binding site of *Tb*GAPDH. Figure 2 depicts the hits and respective data obtained for the NAD^+^ pharmacophores.

### 2.2. Experimental Validation of TbGAPDH Inhibition

To experimentally validate the predictions of the *in silico* screening, a spectrophotometric assay with recombinant *Tb*GAPDH, measuring the increase of NADH during the conversion of 3-phosophoglyceraldehyde into 1,3-bisphosphoglycerate, was used [27]. The increase of absorbance at 340 nm was measured for 1 min after the addition of the substrate. Initially, each test compound was added in a concentration of 50 µM and the slope of the resulting linear plots of absorbance *vs*. time compared to that of a control recorded in the absence of an inhibitor. Out of the 13 hits predicted by pharmacophore screening and molecular docking, nine compounds (69%) were found to significantly inhibit the activity of *Tb*GAPDH. The measured percent inhibition data are reported in Table 1.

Five compounds, namely NP-011694 (**1**), NP-013296 (**2**), NP-013378 (**4**), three geranylated benzophenone derivatives described as constituents of the endophytic fungus *Geniculosporium sp.*, Xylariaceae, NP-013330 (**3**, flavaspidic acid AB found in the fern *Dryopteris crassirhizoma,* Dryopteridaceae), as well as NP-014428 (**5**, a 1,14-bis-resorcinyl-substituted tetradecene derivative from *Grevillea whiteana*, Proteaceae) inhibited the enzyme activity by more than 50% at 50 µM. For each of these five hits, an IC_50_ value was determined. The underlying diagrams showing inhibitory activity as a function of concentration are shown, for each of these compounds, in Supplementary Figures S14–S18, Supplementary Information, and the resulting IC_50_ values are reported in Table 1.

**Table 1:** Experimental data of the compounds tested in the *Tb*GAPDH inhibition assay; data represent inhibition values in % at 50 µM (n.i. = no inhibition at 50 µM) and IC_50_ values in µM concentration.

Compound	% Inhibition at c = 50 µM	IC_50_ (µM)
NP-005755	44	****
NP-007496	n.i.	
NP-008941	n.i.	
NP-010904	n.i.	
NP-011694 (**1**)	66	24.56 ± 1.03
NP-012278	24	
NP-013070	33	
NP-013296 (**2**)	98	4.73 ± 1.04
NP-013330 (**3**)	88	21.97 ± 1.03
NP-013378 (**4**)	>90	6.68 ± 1.04
NP-014428 (**5**)	>90	22.79 ± 1.01
NP-015973	n.i.	
NP-019430	45	

The most active inhibitors, both predicted to bind to the G-3-P site of *Tb*GAPDH, were Compounds **2** and **4**. It is interesting to note that they are structurally very closely related, representing regioisomers in which only the aldehyde and carboxylic acid substituents are exchanged. Compound **1**, in which the aldehyde function of **2** is replaced by a second carboxylic acid moiety, is significantly less active, so that obviously, the presence of an aldehyde function as in the natural substrate leads to enhanced activity.

In comparison to previously-known *Tb*GAPDH inhibitors [18,19,20,21,22], the IC_50_ values of our compounds are quite low, so that these hits obtained from *in silico* screening can be considered quite strong inhibitors of the target enzyme. To our knowledge, the strongest *Tb*GAPDH inhibitor known up to now is a synthetic adenosine derivative with an IC_50_ of 2 µM [17]. The most active inhibitor of natural origin previously known for this enzyme is the diterpene monomethyl kolavate with an IC_50_ value of 12 µM [18], which is about 2.5-fold less potent than our Compound **2**.

### 2.3. Mechanistic Considerations

**Figure 3:**
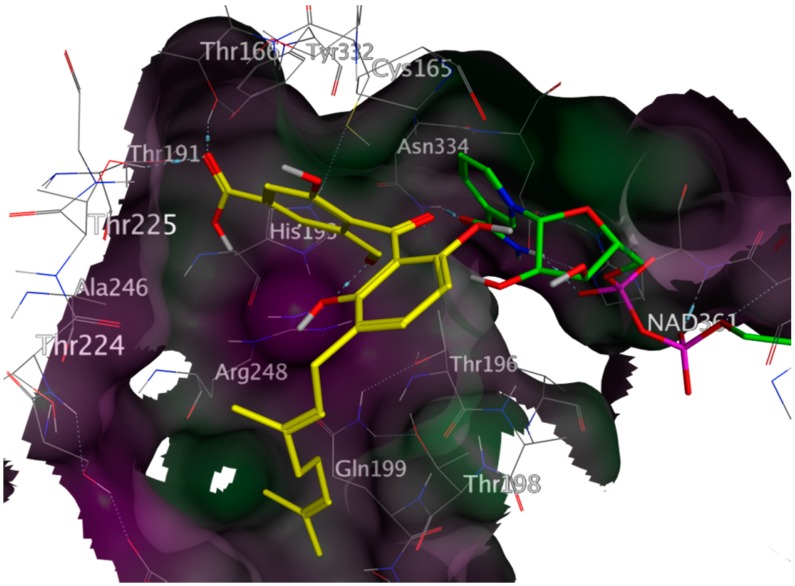
Lowest energy docking pose of compound NP-013296 (**2**) (yellow) in the G-3-P site of *Tb*GAPDH (PDB-ID 2X0N), NAD^+^ shown with green carbon atoms; NP-013296 fits the G-3-P cavity comparably to the natural substrate; hence, it was a good candidate for *in vitro* experiments; rendered surface colored according to lipophilicity, green indicating high, purple low lipophilicity.

**Figure 4:**
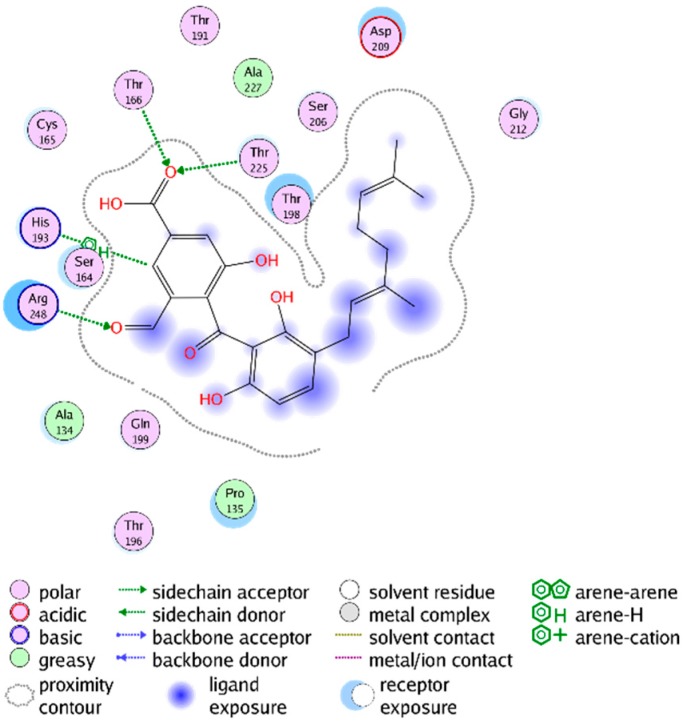
Interactions between the lowest-energy docking pose of Compound **2** (NP-013296) and the G-3-P site of *Tb*GAPDH (PDB-ID 2X0N).

Four of the active inhibitors (**1**–**4**) were identified during the VS using the pharmacophore model for the G-3-P binding site and docking simulations at the corresponding binding site of *Tb*GAPDH. The lowest-energy docking pose of each mentioned compound fit the G-3-P cavity very well. All four compounds showed significantly better docking scores than the natural substrate (S_(**1**)_ = −5.52 kcal/mol; S_(**2**)_ = −6.66 kcal/mol; S_(**3**)_ = −6.12 kcal/mol; S_(**4**)_ = −5.41 kcal/mol; S_G-3-P_ = −3.81 kcal/mol). The docking results also indicated, at least to a certain extent, comparable binding modes of the identified inhibitors and the natural ligand. G-3-P was predicted to show H-bond interactions with Cys 165, Thr 196, Thr 198, Arg 248 and with the cosubstrate NAD^+^. Even though the best docking poses of the inhibitors differ from each other in detail, in all four cases, one of the aromatic moieties of the inhibitors is predicted to extend deeply into the G-3-P binding pocket, where it could interact with various amino acid residues in the immediate vicinity of the catalytic site and would interfere with substrate binding and catalysis. The docking simulation carried out for Compound **1** indicated the formation of an H-bond with Thr 224. Compound **2** was predicted to form H-bonds with His 193, Thr 166, Thr 225 and Arg 248. It represented the strongest *Tb*GAPDH inhibitor found in this study. Inhibitor **3** was calculated to form significant H-bond interactions with Thr 196, Thr 198, Thr 166 and Thr 224. Compound **4** displayed only weak interactions with Cys 165 and Thr 225 (due to the implemented threshold in MOE’s ligand interaction algorithm, these interactions are not shown in Supplementary Figure S12, Supplementary Information), but fit the G-3-P cavity also quite well. Although these predicted binding modes differ from each other, these findings support the hypothesis that all four structurally-related compounds may act as inhibitors competing with G-3-P for the substrate binding site. Figure 3 shows the lowest energy pose of the most active inhibitor **2** docked into the G-3-P binding site, and the resulting ligand interactions are depicted in Figure 4. Analogous plots of Compounds **1**, **3** and **4** are shown in Supplementary Figures S7–S12, Supplementary Information.

**Figure 5:**
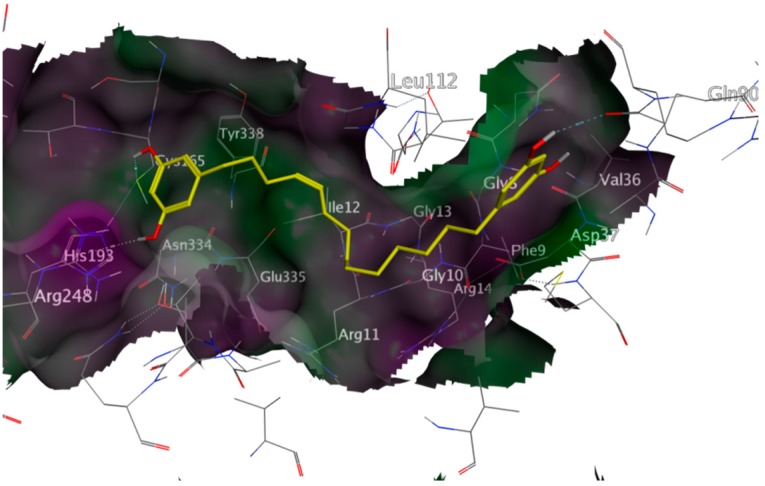
Lowest energy docking pose of compound NP-014428 (**5**) (yellow) in the NAD^+^ binding site of *Tb*GAPDH (PDB-ID 2X0N) (co-crystallized NAD^+^ not shown); rendered surface colored according to lipophilicity, green indicating high, purple low lipophilicity.

**Figure 6:**
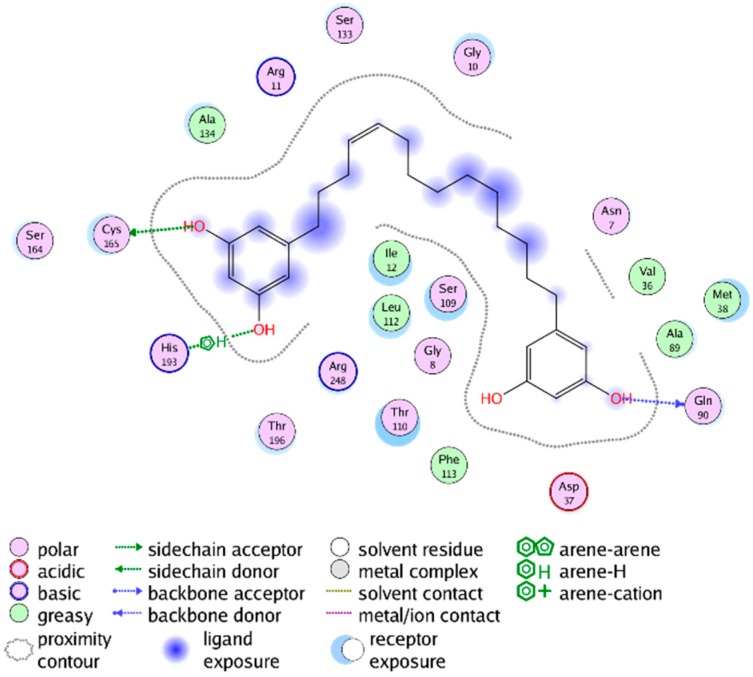
Interactions between the lowest energy docking pose of compound NP-014428 (**5**) and the NAD^+^ binding site of *Tb*GAPDH (PDB-ID 2X0N).

In contrast to Inhibitors **1**–**4**, Compound **5** was identified by the virtual screening with the pharmacophore for the NAD^+^/NADH binding site and docked into this site very well. The lowest energy docking pose of Compound **5** in the NAD^+^/NADH-site of *Tb*GAPDH is shown in Figure 5, and the resulting ligand interactions are depicted in Figure 6. The binding interaction can, in large part, be attributed to steric similarity to NAD^+^ and the relatively large lipophilic part of Compound **5** interacting with the hydrophobic region of the cosubstrate site. Nevertheless, anchoring hydrogen bond interactions are predicted for both resorcinol moieties, namely with Gln 90 in the vicinity of the “adenine end” (right side in Figure 6), but also with Cys 165 and His 193, both of which are actually part of the adjacent G-3-P site (left side of Figure 6) and important for the catalytic mechanism of GAPDH. The docking scores of **5** and the cosubstrate NAD^+^ were −8.10 and −9.10 kcal/mol, respectively. From this result, it might be expected that Inhibitor **5** acts by a competitive mechanism at the NAD^+^/NADH site.

### 2.4. In Vitro Antitrypanosomal Activity of Compounds **1**–**5**

Compounds **1**–**5** were tested for their *in vitro* activity against bloodstream forms of *T. brucei rhodesiense* (causative agent of East African HAT). The results are reported in Table 2. It was found that all of these compounds possess moderate antitrypanosomal activity at an activity level comparable to their *Tb*GAPDH inhibitory potency. Compound **4** with an IC_50_ value of 6.7 µM was the most active against *T. brucei*, and this value is the same as its IC_50_ for *Tb*GAPDH inhibition. Control measurements of cytotoxicity against mammalian cells (L6 rat skeletal myoblasts) revealed that Compounds **2** and **3** are not significantly selective against *T. brucei*, whereas Compounds **3** and **4** do possess a certain degree of selectivity against the parasite (the selectivity index SI = IC_50_(cytotoxicity)/IC_50_(parasite) presented values of three and seven, respectively). On the other hand, Compound **5** was found to be more toxic to the rat cells than to the trypanosomes (SI = 0.5), which might be a consequence of its putative binding to the NAD^+^ site and the possible binding to related binding sites in other enzymes (see above).

**Table 2:** *In vitro* antitrypanosomal and cytotoxic activity of Compounds **1**–**5**. Each value represents the mean ± margin of deviation of two independent determinations. SI, selectivity index.

Compound	*T. brucei rhodesiense* (STIB 900) IC_50_ (µM)	Cytotoxicity (L6) IC_50_ (µM)	SI
NP-011694 (**1**)	39.5 ± 3.1	50.6 ± 3.8	1.3
NP-013296 (**2**)	41.0 ± 0.5	48.7 ± 3.8	1.2
NP-013330 (**3**)	17.9 ± 1.6	54.2 ± 0.5	3.0
NP-013378 (**4**)	6.7 ± 2.2	48.9 ± 4.1	7.3
NP-014428 (**5**)	29.3 ± 6.3	15.5 ± 1.8	0.5
Melarsoprol	0.005 ± 0.001	n.t.	n.d.
Podophyllotoxin	n.t.	0.019 ± 0.002	n.d.

n.t.: not tested; n.d.: not determined.

## 3. Experimental Section

### 3.1. Database Design

The database of commercially available NPs (MEGx rel. 130,901, AnalytiCon Discovery GmbH, Potsdam, Germany) consisted of 4803 natural products of various origins. This database was filtered according to Lipinski’s rule of 5 to eliminate non-drug-like compounds and then reduced to compounds without chiral centers in order to avoid the problems associated with undefined or misassigned stereocenters. The resulting subset of compounds (700 in total) was subjected to full geometry optimization using the MMFF94x force field [28] and the low mode molecular dynamics (LowModeMD) [29] conformational search algorithm (with standard settings). The calculated ten most favorable conformations of each compound were collected into a database and served as a working database for the virtual screening protocol.

### 3.2. Acquisition and Preparation of the Proteins’ 3D-Structure

The 3D-structures of glyceraldehyde-3-phosphate-dehydrogenases from *Trypanosoma brucei brucei* (PDB-ID 2X0N, resolved to 3.2 Å by X-ray diffraction), *Trypanosoma cruzi* (PDB-ID 3IDS, resolved to 1.80 Å by X-ray diffraction) and *Leishmania mexicana* (PDB-ID 1GYP, resolved to 2.80 Å by X-ray diffraction) were retrieved from the Protein Data Bank [25]. The structures were corrected (“Structure Preparation” in MOE, correcting, e.g. terminal amino acids), protonated (“Protonate 3D” algorithm in MOE) and energy minimized employing force field techniques (series of energy minimizations with heavy atoms tethered with force constants, subsequently, 100, 10, 1, 0.1 and 0, MMFF94x force field). A self-docking of the given co-crystallized ligands was performed in MOE (induced fit, MMFF94x force field, placement by implemented triangle matcher, rescoring via the London dG algorithm, best poses refined by force field calculations and rescored via GBVI/WSA dG, retaining at least 10 poses for each calculation) yielding an S-score given in kcal/mol. This S-value constituted the basis for the identification of new lead structures, indicating that a similar or even more negative docking score of an examined compound in comparison to the natural ligand represents a good hint for a possible inhibitory activity.

### 3.3. Pharmacophore Design and Virtual Screening

In order to perform a virtual screening with the NP database mentioned above, pharmacophore queries were created for each of the enzymes mentioned above employing the software package MOE. These pharmacophore schemes served as the main filter for the virtual screening (VS) performed on the natural product database. Due to a highly conserved, but still slightly differing 3D-structure of the mentioned enzymes and co-crystallized ligands, pharmacophores originating from the NAD-binding site of different trypanosomatid parasites’ GAPDHs (*L. mexicana*, *T. brucei* and *T. cruzi*) were used for the VS. Two pharmacophore queries were created for *Tb*GAPDH, one for the 3-PGA and one for the NAD^+^ site.

First of all, an interaction table of the active site of the enzyme of interest and its co-crystallized ligand was created using the “ligand interactions” feature implemented in MOE. Every interaction yielding a calculated S-score less than −1.0 kcal/mol was considered to be of relevance and was therefore included in the pharmacophore model as a feature.

The diameter of each pharmacophore sphere was set to values between 1.5 and 2 Å, depending on the size of the moiety of interest. Additionally, exclusion volumes were defined, ruling out compounds that collided or interfered with amino acids of the enzymes’ active site by means of their size. The exclusion volumes were obtained by creating an exclusion sphere around every atom of the protein (solvent molecules excluded) with a given diameter as suggested by MOE (e.g., 1.2 Å for hydrogen, 1.4 Å for oxygen, 1.54 Å for nitrogen and 1.85 Å for carbon atoms). These efforts resulted in pharmacophore queries ranging from about 5–10 features depending mainly on the size of the explored pocket and the interactions mediated by the co-crystallized ligand.

**Figure 7:**
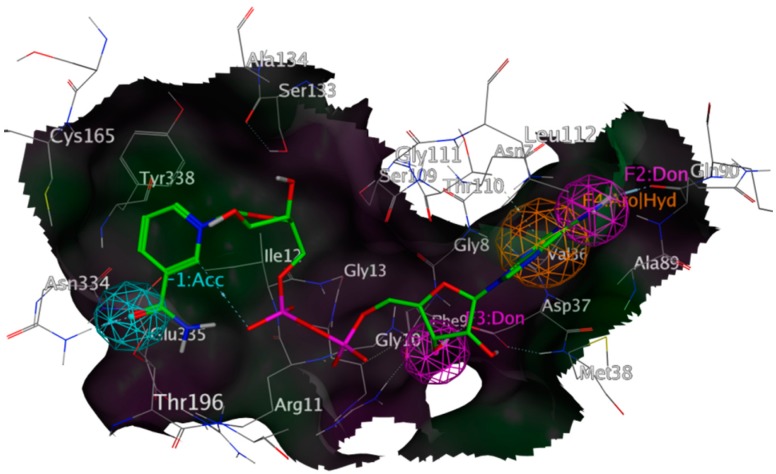
Representation of the pharmacophore based on an NAD^+^ binding site from *Tb*GAPDH (PDB-ID 2X0N), co-crystallized NAD^+^ shown with green carbon atoms; rendered surface colored according to lipophilicity, green indicating high, purple low lipophilicity; aromatic features in orange; H-bond donor features in purple; H-bond acceptor features in cyan. Interactions employed as features were calculated with MOE (“ligand interactions” algorithm); exclusion spheres are hidden.

The predictive value of the created pharmacophores was evaluated by using each query for the identification of the co-crystallized ligand on which the pharmacophores’ design was based (except the pharmacophore for the G-3-P site). A query was considered to be effective if it identified the given conformation of the co-crystallized ligand as a potential hit. With this requirement met, the pharmacophore schemes were used for the screening of the natural product databases. In order to increase the rate of identified NPs for further *in silico* and *in vitro* investigations, compounds fitting the pharmacophores not completely, but to a predefined extent (partial match), were also considered to be relevant. The structures of the resulting hits were collected into a new database, which was then submitted to molecular docking. A representation of the *Tb*GAPDH pharmacophore is given in Figure 7; the corresponding pharmacophores of *Lmex*GAPDH, *Tc*GAPDH and the G-3-P pharmacophore for *Tb*GAPDH are depicted in Supplementary Figures S1–S3, Supplementary Information. A set of figures allowing the comparison of the active sites of all three mentioned enzymes is also given (Supplementary Figures S4–S6).

### 3.4. Docking Procedures

The identified hits returned by the diverse pharmacophores were subjected to molecular docking simulations. Initially, a rigid docking (*i.e*., protein and ligand structures were treated as rigid) into the respective enzyme structure was performed with all hits returned from each VS query using the docking algorithm implemented in MOE (placements and scorings according to the self-docking procedure mentioned above). The output databases were ranked according to the calculated S-scores. The ten compounds yielding the most negative S-score from each of the rigid docking simulations with the various enzymes/binding sites were then combined and docked into the appropriate binding site of *Tb*GAPDH applying an induced fit docking algorithm (*i.e*., both the binding site and ligand treated as flexible; placements and scorings according to the procedures mentioned above). This more precise docking procedure returned a more accurate ranking of the docked ten compounds, out of which the best five (ranked by most negative S-score) were taken into account for *in vitro* testing (compounds identified in duplicate by the diverse pharmacophores were removed, which resulted in a final test set of 13 compounds).

All files used for the *in silico* part of this study (protein and ligand structures, as well as pharmacophore query definitions) are available from the authors on request.

### 3.5. Tested Compounds

All compounds tested experimentally were obtained from AnalytiCon Discovery GmbH, Potsdam, Germany. Their purity was specified by the supplier as determined by NMR and/or HPLC analyses to be >95% in the case of Compounds **1**–**3** as well as **5**, while Compound **4** was specified >70% pure.

### 3.6. Recombinant Expression and Purification of Trypanosoma brucei GAPDH in E. coli

*T. brucei* GAPDH was expressed recombinantly in *E. coli* strain BL21 (DE3) kindly provided by M.L. Bolognesi and S. Piretti (Bologna, Italy) and P. Michels (Edinburgh, U.K.). An overnight culture of the mentioned strain (incubation for 16 h in 25 mL LB medium, 37 °C, supplemented with kanamycin (50 µg/mL)) was used to inoculate Erlenmeyer flasks filled with 400 mL LB medium also supplemented with kanamycin (50 µg/mL). After reaching an optical density of 0.6 to 0.8 (incubation between 4–6 h at 37 °C), the expression of *Tb*GAPDH was induced applying 0.4 mM isopropyl-β-d-thiogalactopyranoside (IPTG). The incubation was continued at 22 °C for 12 to 14 h. Bacteria were harvested afterwards by centrifugation (10 min, 4 °C, 7000 rpm). The pellet was resuspended in 15 mL buffer (100 mM NaH_2_PO_4_ × H_2_O, 50 mM NaCl, 1 mM NAD^+^), and lysozyme (1 mg/mL), phenylmethylsulfonyl fluoride (1 mM) and benzamidine (1 mM) were added. After an incubation of 15 min on ice, an ultrasonic lysis of the *E. coli* material was performed. Cell fragments were removed by further centrifugation (15 min at 15,000 rpm, 4 °C). The supernatant was cleared by double filtration through a 0.45-µm syringe filter and applied to a Ni-chelating column. *Tb*GAPDH was eluted using a gradient of imidazole solutions ranging from 100 mM–500 mM in concentration. The resulting fractions were analyzed using SDS-PAGE, and fractions containing the cleaned protein of interest were joined. Thrombin (1 U/mg protein) was added to cleave the histidine tag. Dialysis was performed afterwards to remove small molecular impurities. The solution was concentrated employing a centrifugation with an AMICON filter (10 min at 7500 rpm, 4 °C), and the concentration and activity of *Tb*GAPDH, as well as its saturation conditions were determined by optical spectroscopy (c_*Tb*GAPDH_ = 7.17 µg; activity of 1 U/244 µg *Tb*GAPDH, tested in presence of 1.5 mM NAD and 0.5 mM G-3-P at 30 °C; saturating concentrations of NAD^+^ = 1.5 and of G-3-P = 0.5 mM, respectively; determination of saturating conditions depicted in Supplementary Figure S13).

### 3.7. T. brucei Glyceraldehyde-3-phosphate Dehydrogenase Inhibition Assay

In order to determine the activity of *Tb*GAPDH, a spectrophotometric assay was used as described before [27]. For that purpose, the increasing amount of produced NADH was measured at 340 nm with a Hitachi U-2900 UV-VIS-Spectrophotometer as a linear indicator for the activity of the catalytic protein. The activity determined without the addition of an inhibitor was set to 100%; the measured inhibitions were calculated accordingly. The assay was performed at 30 °C, in a buffer of 0.1 M TEA, 0.1 M glycine and 0.1 M EDTA with varying concentrations of the tested compounds in a total volume of 1 mL containing 7.17 µg *Tb*GAPDH, 1.5 mM NAD^+^ and 0.5 mM G-3-P. The tested inhibitors were solved in DMSO, resulting in a final concentration of 0.1%–1% of solvent in the assay solution. This concentration of DMSO did not show inhibitory effects itself in the mentioned assay. Typically, 5–7 different concentrations were measured (each of them in triplicate) to determine IC_50_ values, which were calculated using the software “Prism 3.00” (GraphPad) via nonlinear regression analysis. The measured concentration/effect curves are shown in Supplementary Figures S14–S18.

## 4. Conclusions

In the presented *in silico* and *in vitro* study, we identified five new *Tb*GAPDH inhibitors out of a large number of chemically-diverse natural products. The high rate of experimentally-confirmed inhibitors among the *in silico* hits confirms the validity of the employed VS/docking approach. All of the identified compounds are reported to inhibit *Tb*GAPDH for the first time, revealing possible options for further innovative inhibitor design based on their structural properties. The docking results indicate that the identified NPs address two different binding sites, namely the substrate (Compounds **1**–**4**) and the cosubstrate (Compound **5**) binding cavities of the enzyme. At present, it can only be hypothesized on the grounds of this theoretical prediction that they may compete with the natural substrates for these binding sites. Investigations of the inhibition kinetics are warranted.

Taken together, the VS approach applied here, based on pharmacophore hypotheses for the substrate, as well as the cosubstrate site, led to the identification of two different types of inhibitors of the target enzyme with considerable activities. It is also worth mentioning that the pharmacophore for the G-3-P site, even though it was established without analysis of the interactions of a co-crystallized substrate and based only on the analysis of electrostatic maps, led to promising hits with proven *in vitro* activity.

The fact that the enzyme inhibitors found in the present study were also active against *T. brucei rhodesiense* in a cellular assay and in a similar concentration range as required for enzyme inhibition indicates that inhibition of GAPDH may indeed be a major mechanism of their antitrypanosomal activity.

Even though the overall level of activity against *T. brucei rhodesiense* in the cellular assay is at best moderate, the structures of these compounds can be considered interesting starting points for optimization and, possibly, further development. Studies in this direction, along with continued efforts to use the approach described here on further parasite enzymes and NP databases, are in progress.

## Supplementary Materials

Supplementary materials can be accessed at: http://www.mdpi.com/1420-3049/20/09/16154/s1.
