# Supplementary material for: In Silico Identification and in Vitro Activity of Novel Natural Inhibitors of Trypanosoma brucei Glyceraldehyde-3-phosphate-dehydrogenase
Source: Molecules. 2015 Sep 3;20(9):16154–69. doi: 10.3390/molecules200916154 (PMC6332274; doi:10.3390/molecules200916154)
Supplement: Supplementary file 1 [file molecules-20-16154-s001.pdf]

Supplementary Information

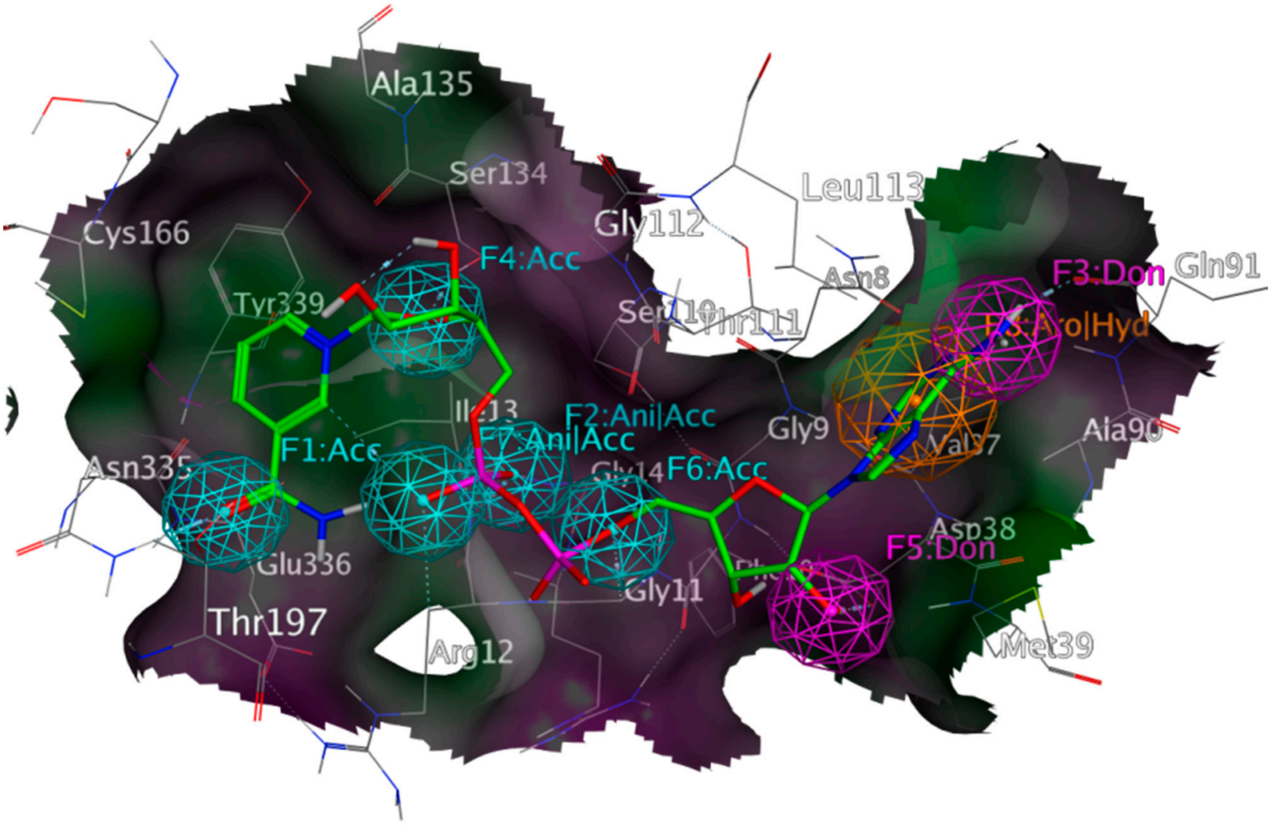

Figure S1. Representation of the pharmacophore based on an NAD^+ binding site from *Lmex*GAPDH (PDB-ID 1GYP), co-crystallized NAD^+ shown with green carbon atoms; rendered surface colored according to lipophilicity, green indicating high, purple low lipophilicity; aromatic features in orange, H-bond donor features in purple, H-bond acceptor features in cyan. Interactions employed as features were calculated with MOE (“ligand interactions” algorithm); exclusion spheres are hidden, non-polar hydrogens not shown. Note the absence of solvent molecules as well as the multiple interactions between the protein and negatively charged oxygen atoms from the diphosphate moiety. Compared to the *Tb*GAPDH pharmacophore for NAD^+, different interactions between the ribose moieties and the active site were also detected by MOE.

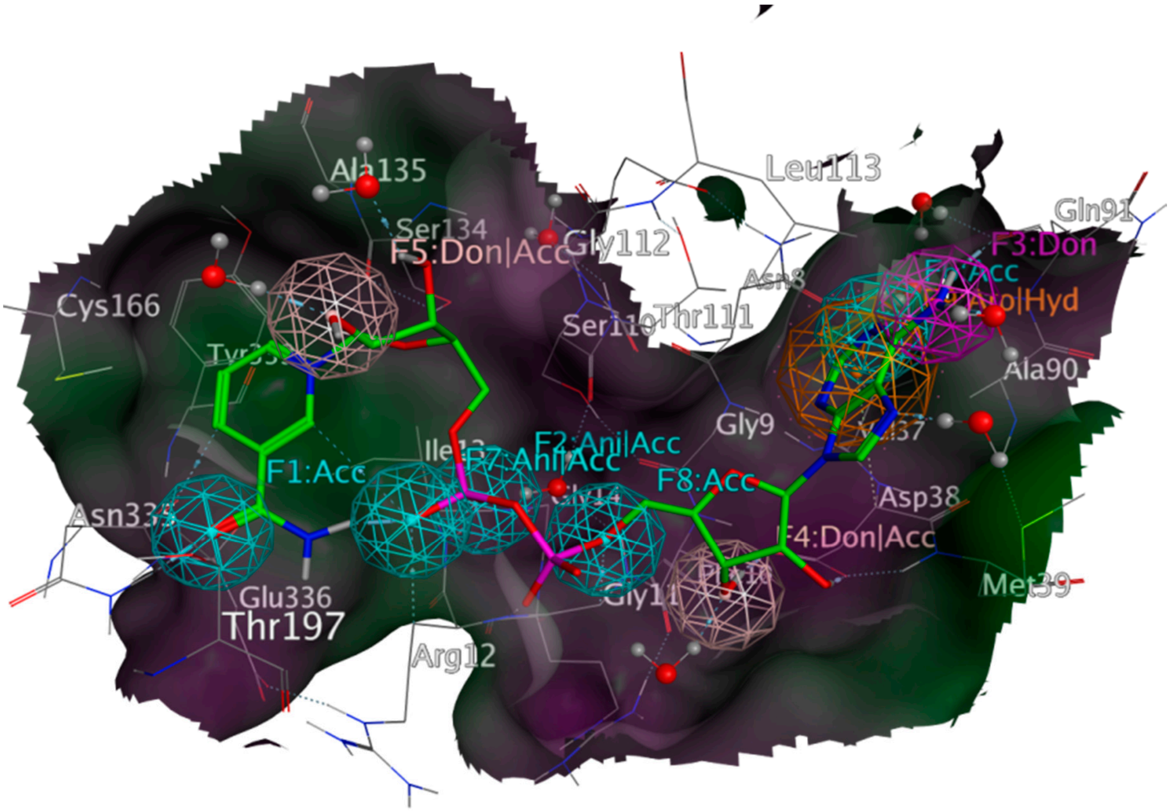

Figure S2. Representation of the pharmacophore based on an NAD^+ binding site from *Tc*GAPDH (PDB-ID 3IDS), co-crystallized NAD^+ shown with green carbon atoms; rendered surface colored according to lipophilicity, green indicating high, purple low lipophilicity; aromatic features in orange, H-bond donor features in purple, H-bond acceptor features in cyan. Interactions employed as features were calculated with MOE ("ligand interactions" algorithm); exclusion spheres are hidden, non-polar hydrogens not shown. Note the presence of co-crystalized solvent molecules mediating interactions with different amino acids of the active site as well as the multiple interactions between the protein and negatively charged oxygen atoms from the diphosphate moiety. Compared to the *Tb*GAPDH pharmacophore for NAD^+, different interactions between the ribose moieties and the active site were also detected by MOE.

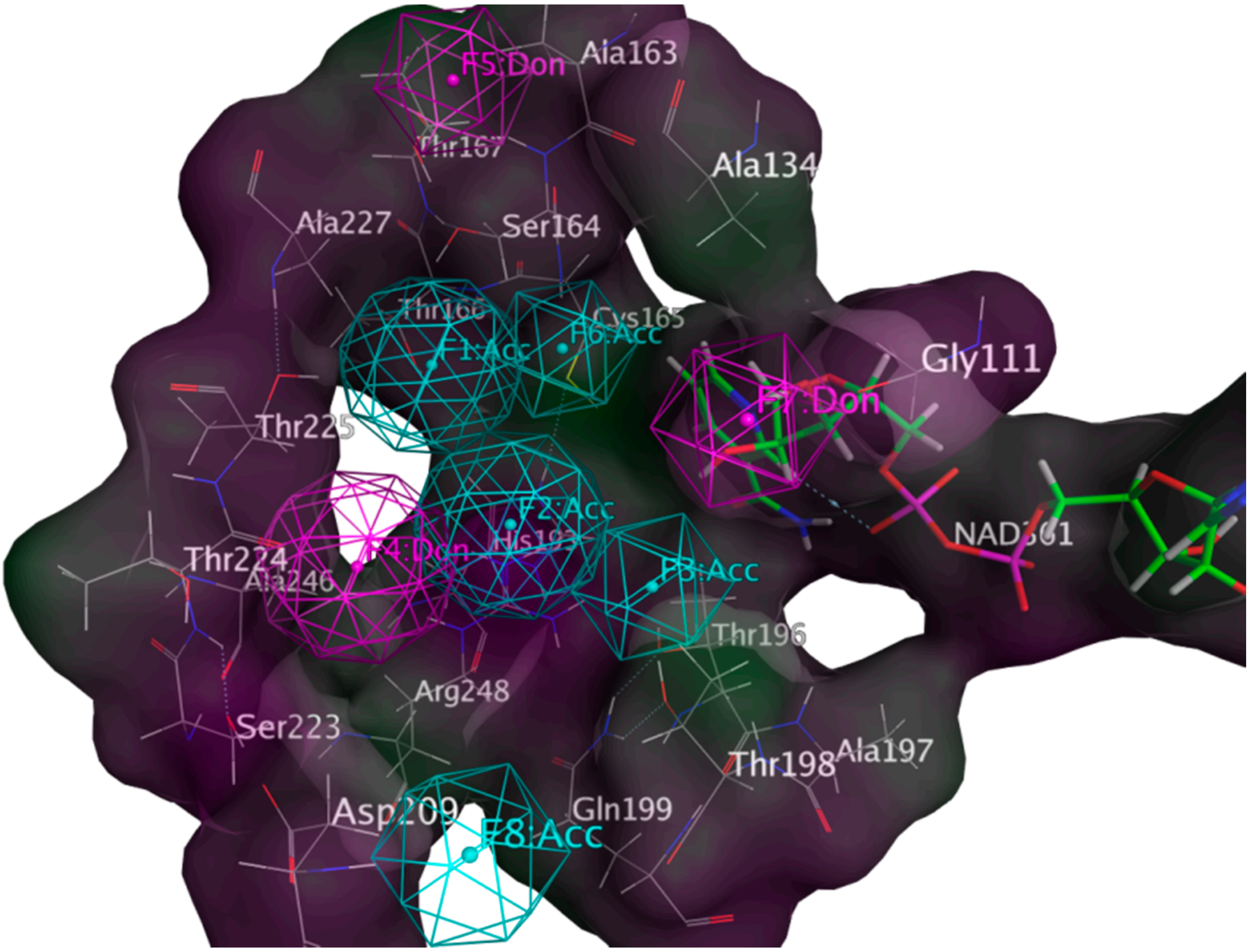

Figure S3. Representation of the pharmacophore based on the G-3-P-site from *Tb*GAPDH (PDB-ID 2X0N), co-crystallized NAD^+ shown with green carbon atoms; rendered surface colored according to lipophilicity, green indicating high, purple low lipophilicity; aromatic features in orange, H-bond donor features in purple, H-bond acceptor features in cyan, exclusion spheres are hidden, non-polar hydrogens not shown. Interactions employed as features were determined by the "electrostatic map" algorithm implemented in MOE due to the absence of a co-crystalized ligand; exclusion spheres are hidden.

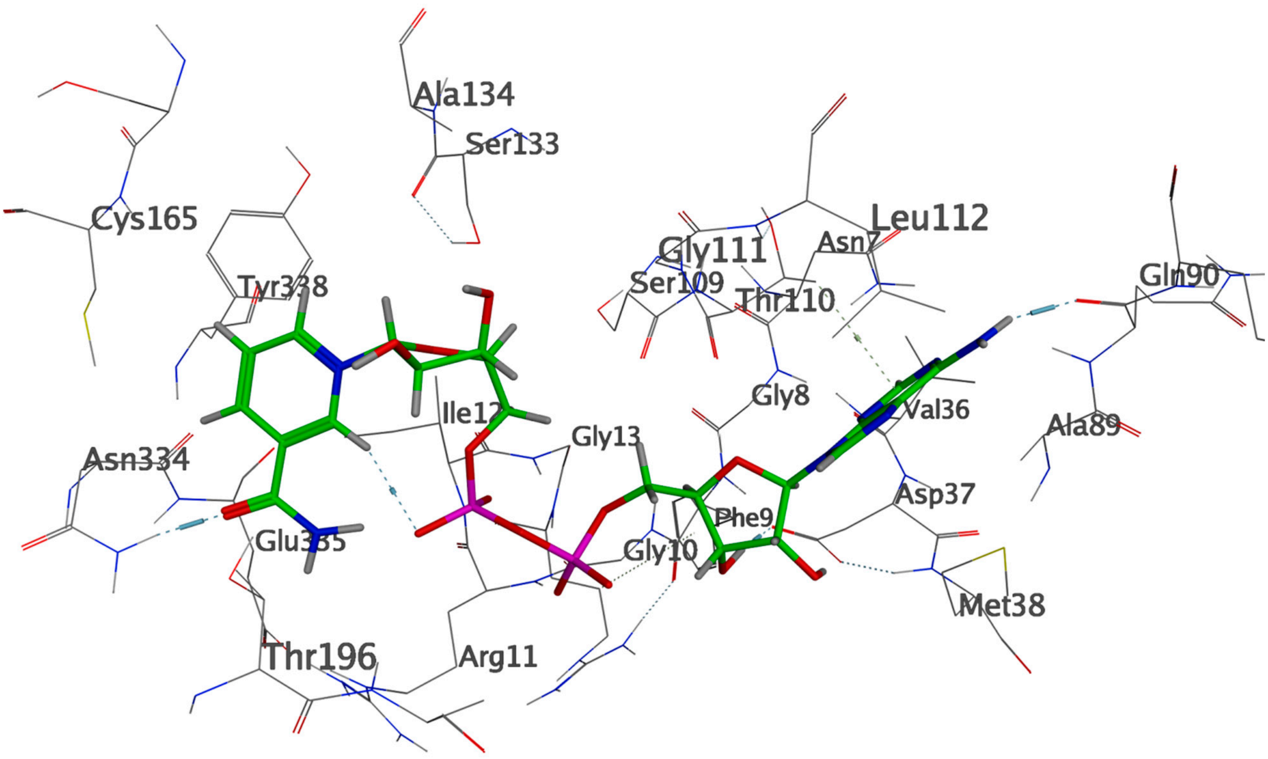

Figure S4. Depiction of the NAD$^+$ site from *Tb*GAPDH (PDB-ID 2X0N). Co-crystallized NAD$^+$ shown with green carbon atoms, non-polar hydrogens not shown.

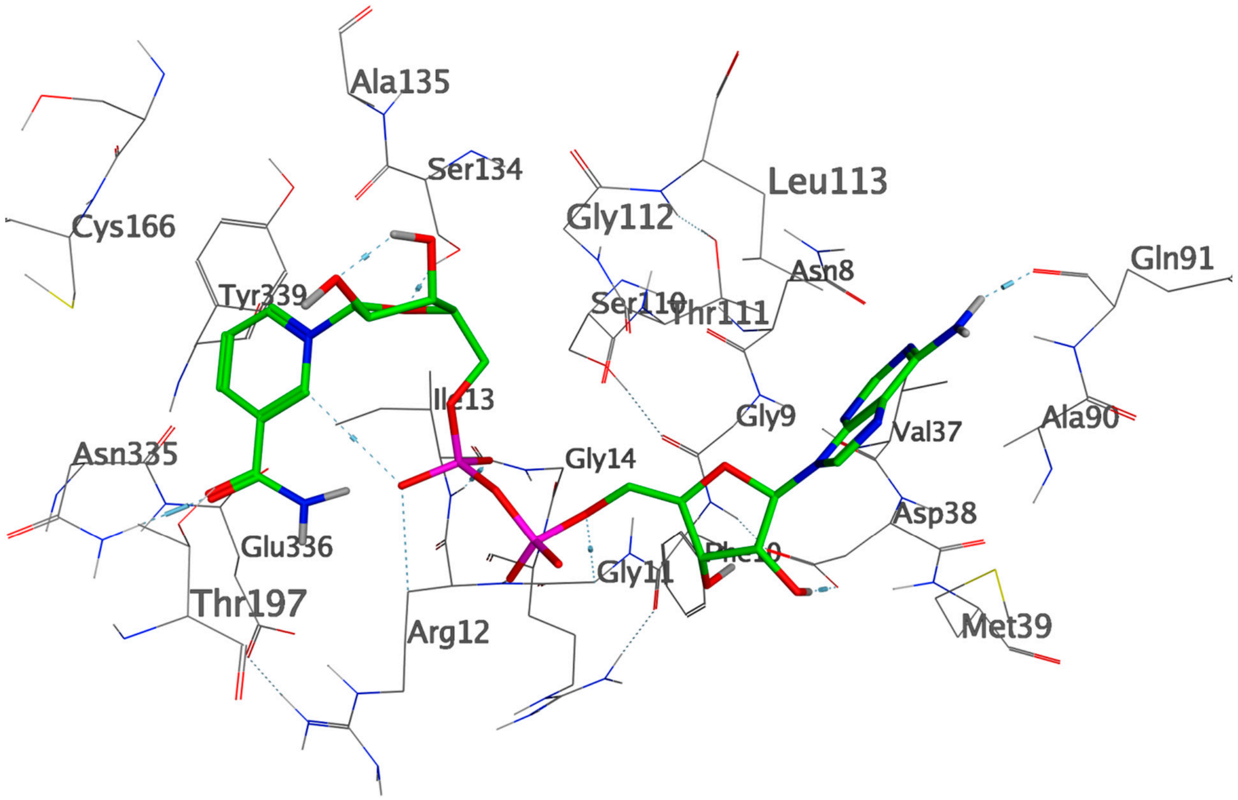

Figure S5. Depiction of the NAD$^+$ site from *Lmex*GAPDH (PDB-ID 1GYP). Co-crystallized NAD$^+$ shown with green carbon atoms, non-polar hydrogens not shown.

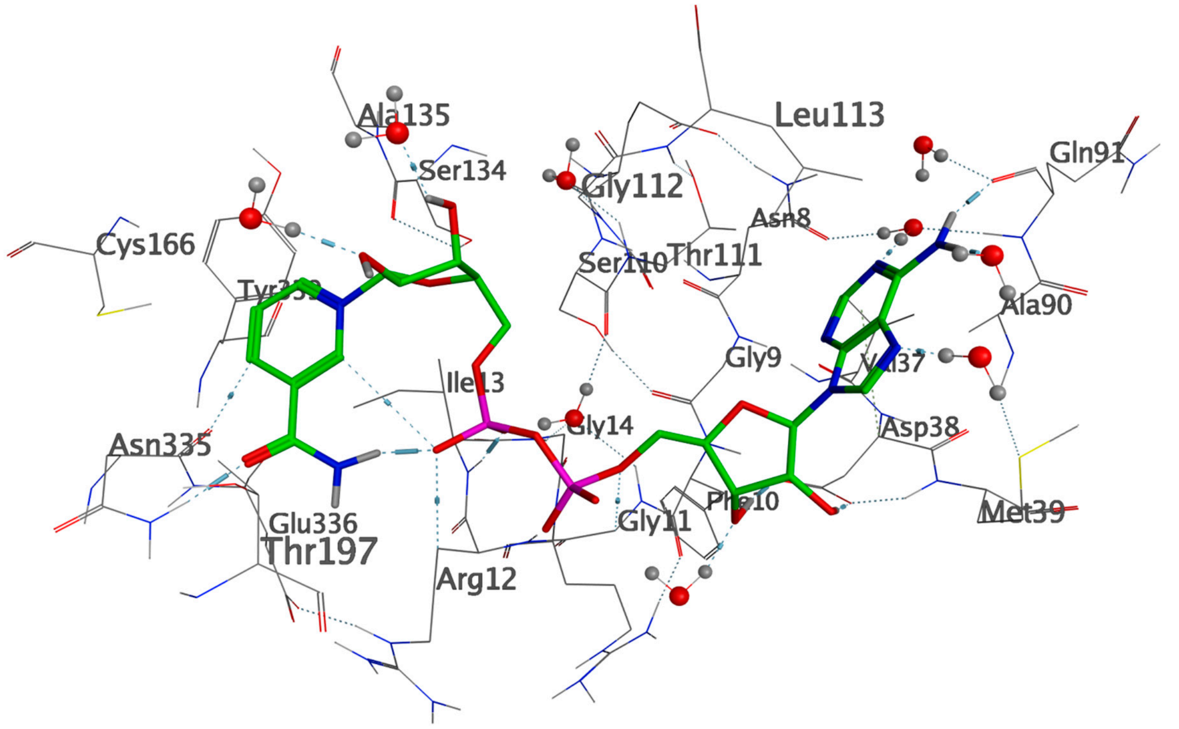

Figure S6. Depiction of the NAD$^+$ site from *Tc*GAPDH (PDB-ID 3IDS). Co-crystallized NAD$^+$ shown with green carbon atoms, non-polar hydrogens not shown.

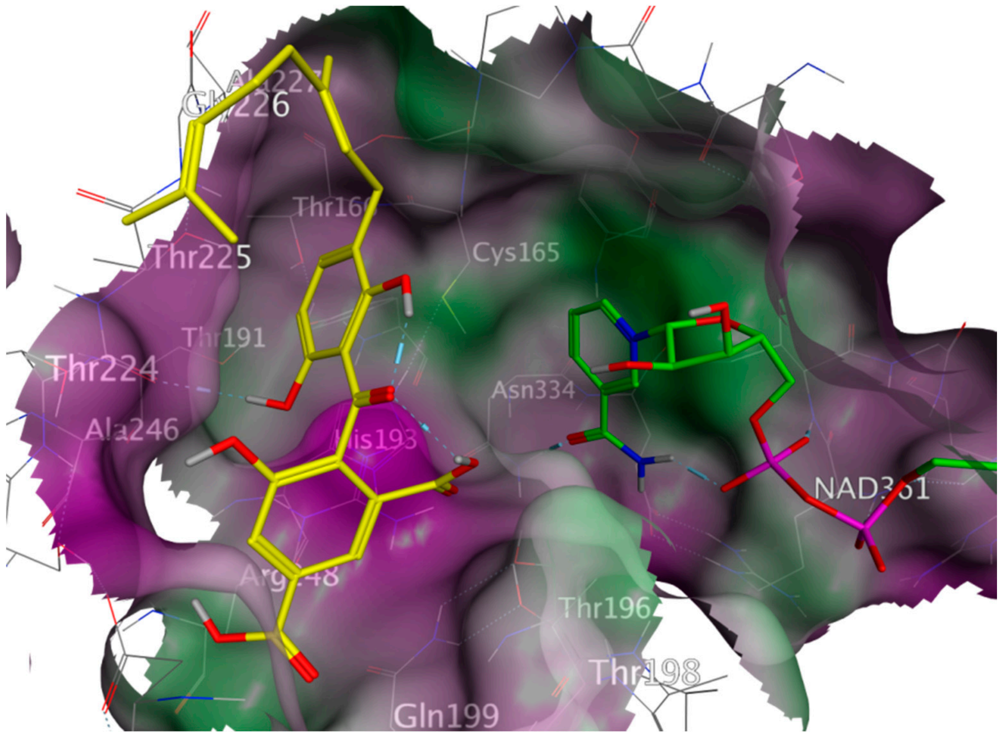

Figure S7. Lowest-energy docking pose of compound NP-011694 (**1**) (yellow) in the G-3-P site of *Tb*GAPDH (PDB-ID 2X0N), co-crystallized NAD$^+$ shown with green carbon atoms; rendered surface colored according to lipophilicity, green indicating high, purple low lipophilicity, non-polar hydrogens not shown.

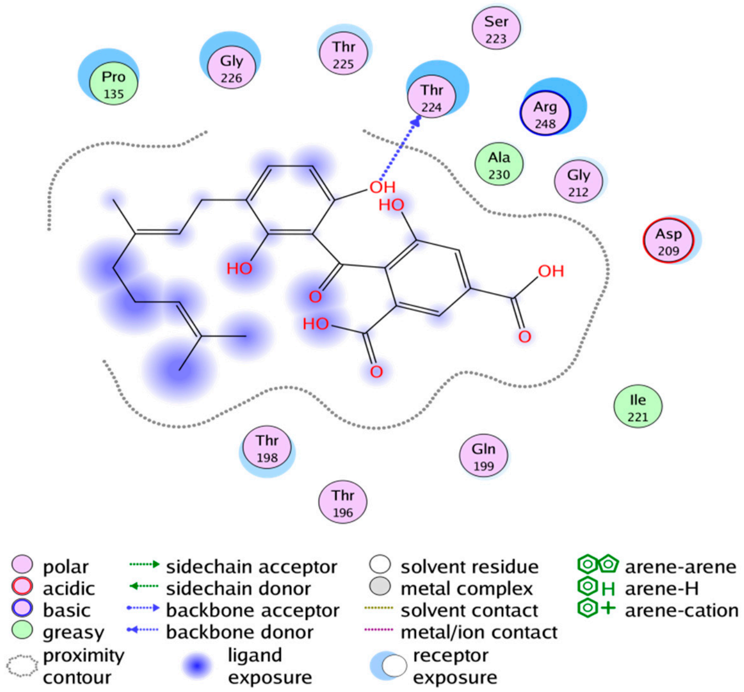

Figure S8. Interactions between the lowest-energy docking pose of compound NP-011694 (**1**) and the G-3-P site of *Tb*GAPDH (PDB-ID 2X0N).

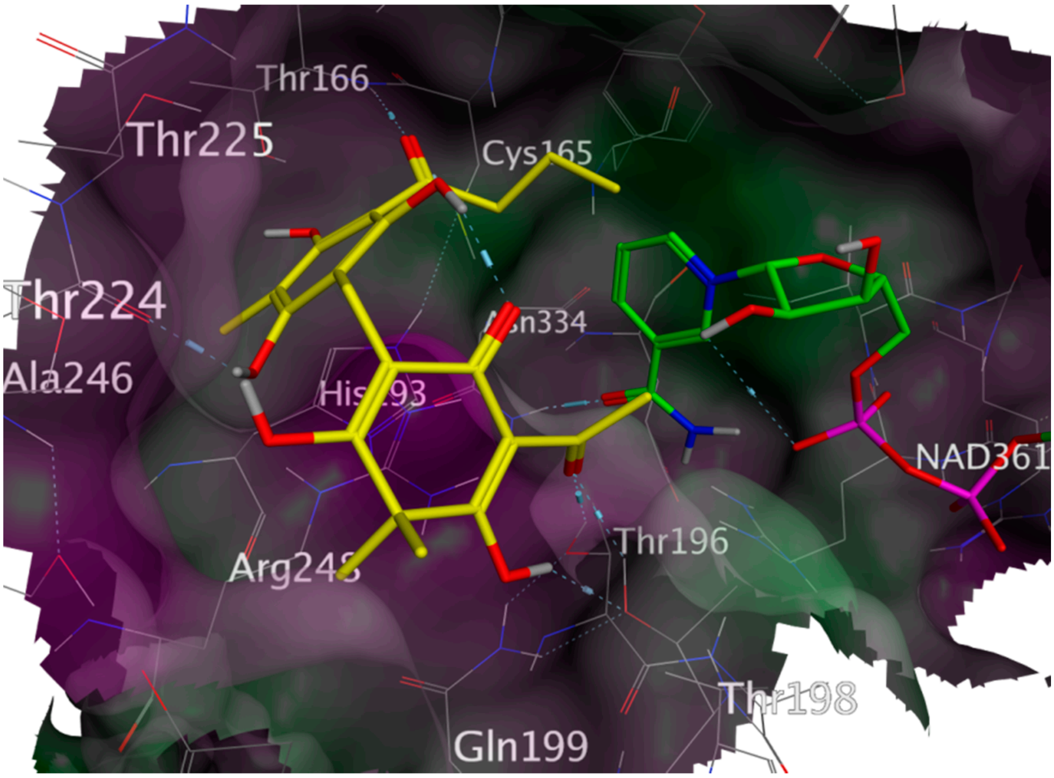

Figure S9. Lowest-energy docking pose of compound NP-013330 (**3**) (yellow) in the G-3-P site of *Tb*GAPDH (PDB-ID 2X0N), co-crystallized NAD^+ shown with green carbon atoms; rendered surface colored according to lipophilicity, green indicating high, purple low lipophilicity, non-polar hydrogens not shown.

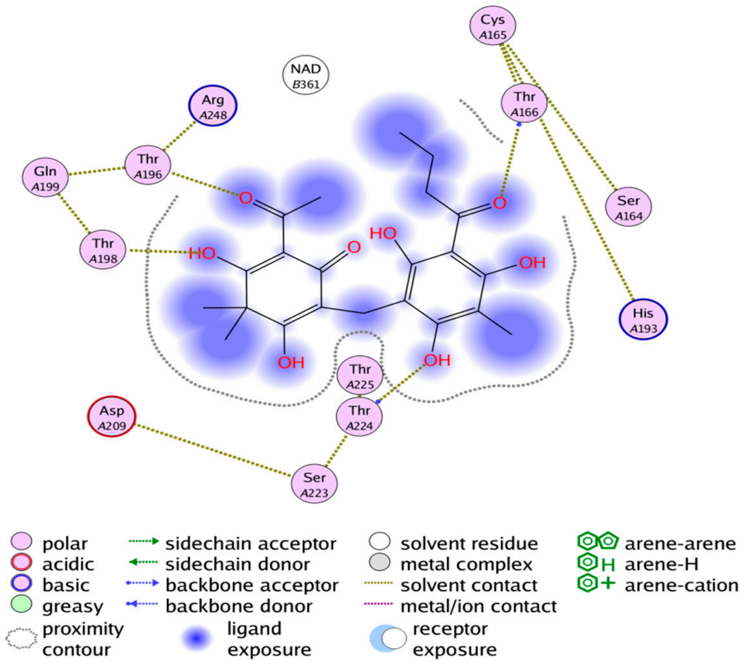

Figure S10. Interactions between the lowest-energy docking pose of compound NP-013330 (**3**) and the G-3-P site of *Tb*GAPDH (PDB-ID 2X0N).

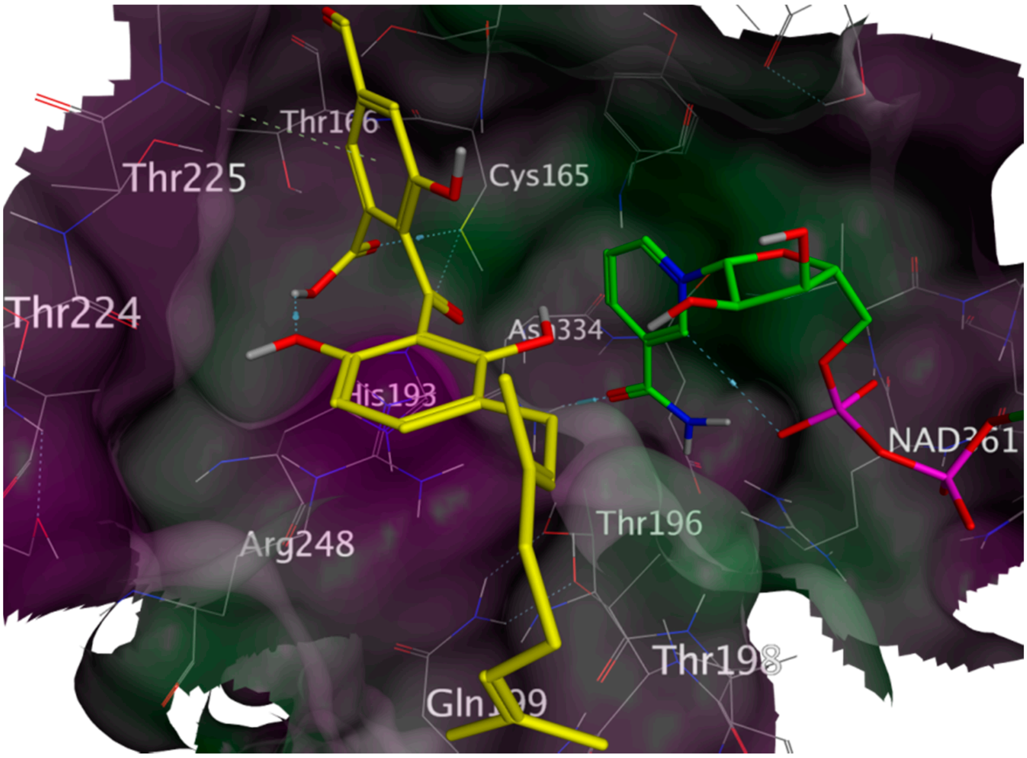

Figure S11. Lowest-energy docking pose of compound NP-013378 (**4**) (yellow) in the G-3-P site of *Tb*GAPDH (PDB-ID 2X0N), co-crystallized NAD$^+$ shown with green carbon atoms; rendered surface colored according to lipophilicity, green indicating high, purple low lipophilicity, non-polar hydrogens not shown.

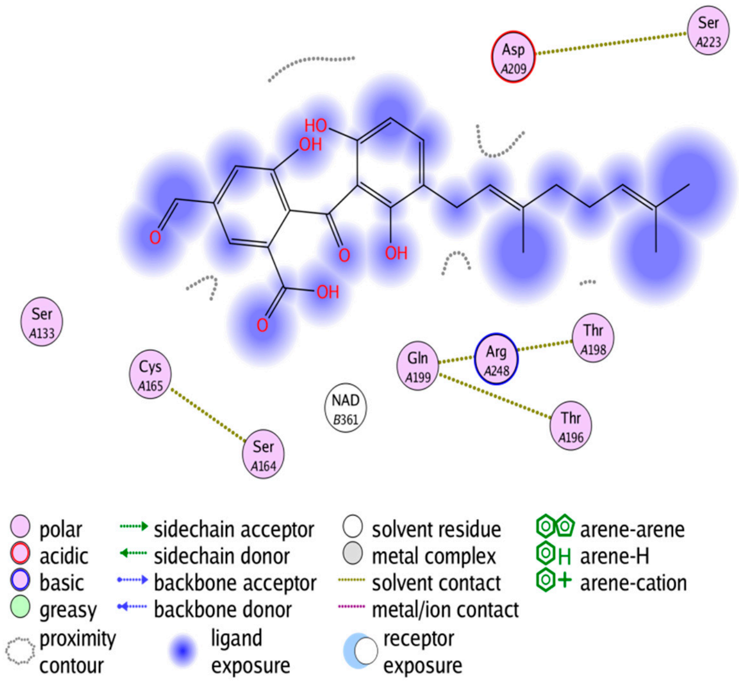

Figure S12. Interactions between the lowest-energy docking pose of compound NP-013378 (**4**) and the G-3-P site of *Tb*GAPDH (PDB-ID 2X0N).

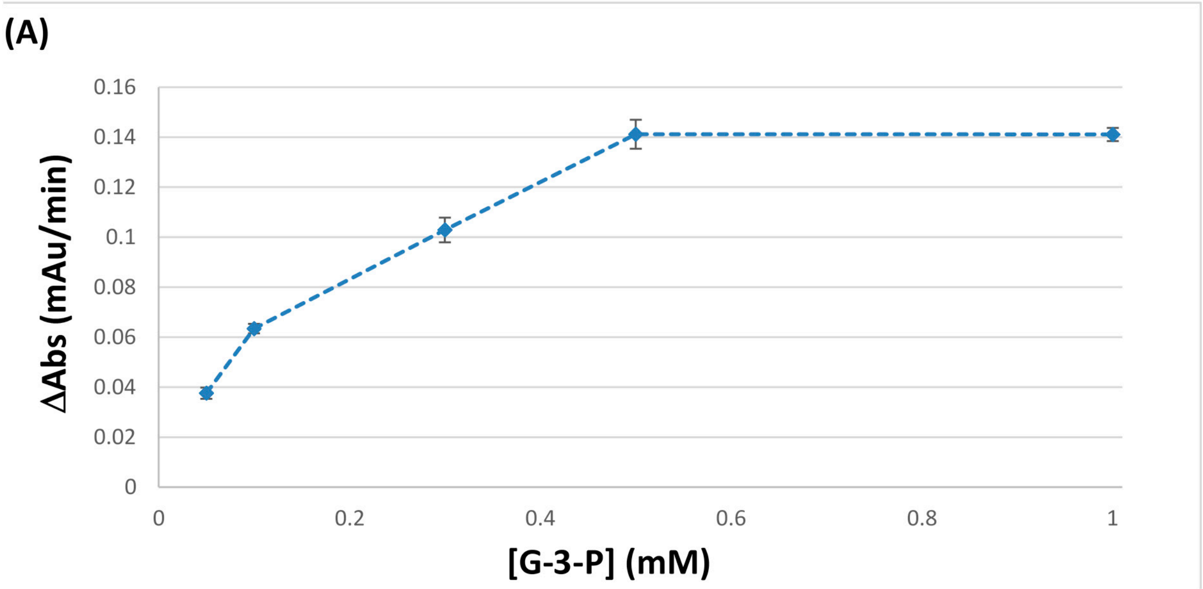

Figure S13. *Cont.*

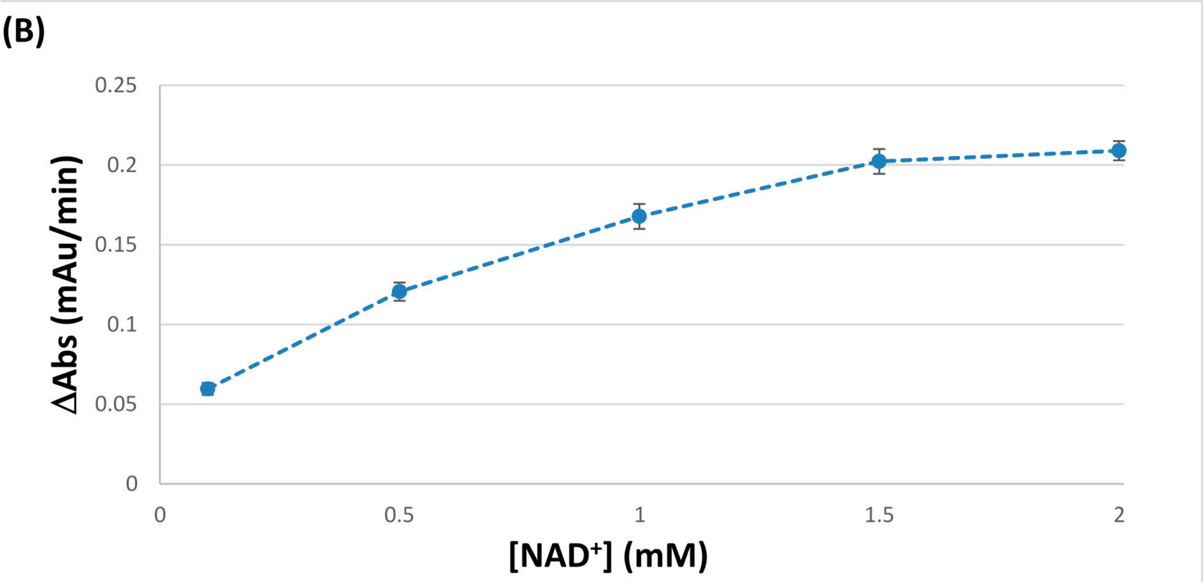

Figure S13. Experimental determination of *Tb*GAPDH´s saturating conditions of G-3-P and NAD^+; The extent of enzymatic conversion was monitored by following the increase of absorbance at 340 nm as a linear kinetic parameter. (**A**) Co-Substrate NAD^+ in excess (2 mM) while varying concentrations of G-3-P from 0.1 to 1 mM were used; (**B**) Substrate G-3-P in excess (1 mM) while varying concentrations of NAD^+ from 0.1 to 2 mM were used.

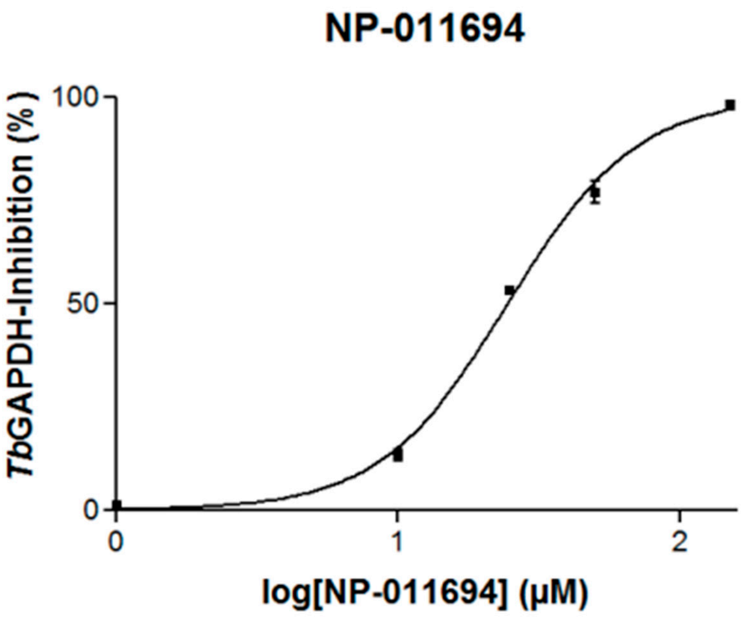

Best-fit values	
BOTTOM (Constant)	0.0
TOP (Constant)	100.0
LOGEC50	1.390
HILLSLOPE	1.906
EC50	24.56
Std. Error	
LOGEC50	0.01241
HILLSLOPE	0.1028
95% Confidence Intervals	
LOGEC50	1.363 to 1.417
HILLSLOPE	1.684 to 2.128
EC50	23.09 to 26.13
Goodness of Fit	
Degrees of Freedom	13
R^2	0.9940
Absolute Sum of Squares	121.8
Sy.x	3.061
Data	
Number of X values	5
Number of Y replicates	3
Total number of values	15
Number of missing values	0

Figure S-14. IC_{50}-Determination of compound NP-011694 (**1**). The absolute IC_{50}-value was determined by nonlinear regression analysis employing GraphPad Prism 3.00 and is given in Table 1.

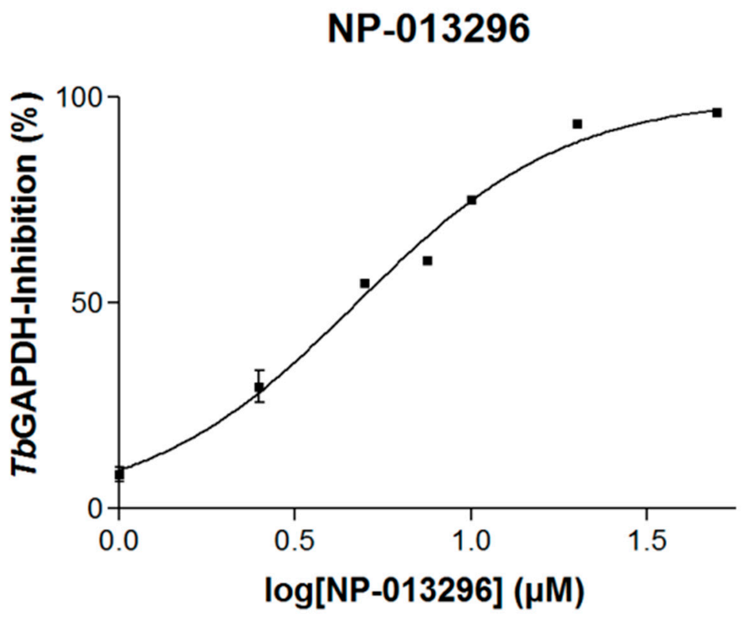

Best-fit values	
BOTTOM (Constant)	0.0
TOP (Constant)	100.0
LOGEC50	0.6752
HILLSLOPE	1.460
EC50	4.733
Std. Error	
LOGEC50	0.01580
HILLSLOPE	0.08073
95% Confidence Intervals	
LOGEC50	0.6421 to 0.7082
HILLSLOPE	1.291 to 1.629
EC50	4.386 to 5.108
Goodness of Fit	
Degrees of Freedom	19
R^2	0.9835
Absolute Sum of Squares	314.4
Sy.x	4.068
Data	
Number of X values	7
Number of Y replicates	3
Total number of values	21
Number of missing values	0

Figure S15. IC_{50}-Determination of compound NP-013296 (**2**). The absolute IC_{50}-value was determined by nonlinear regression analysis employing GraphPad Prism 3.00 and is given in Table 1.

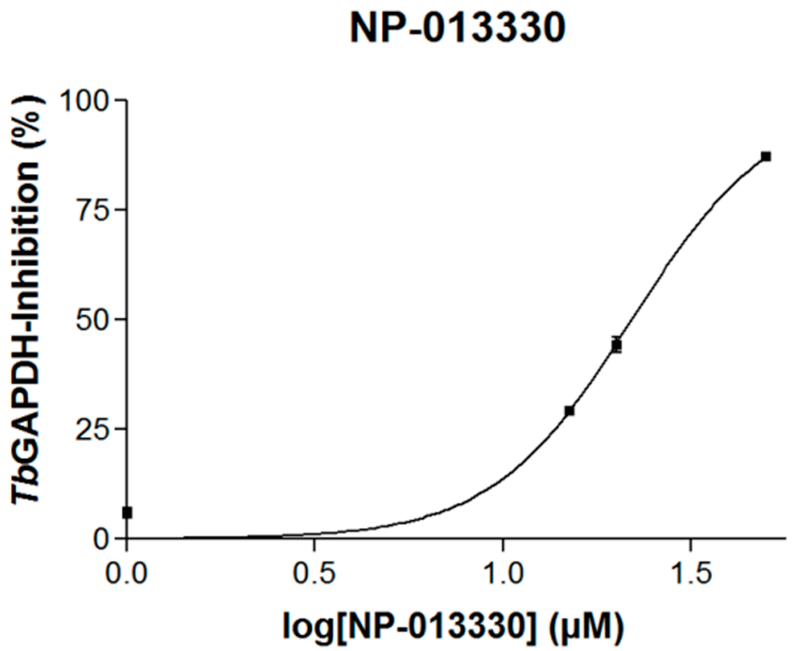

Best-fit values	
BOTTOM (Constant)	0.0
TOP (Constant)	100.0
LOGEC50	1.342
HILLSLOPE	2.324
EC50	21.97
Std. Error	
LOGEC50	0.01212
HILLSLOPE	0.1790
95% Confidence Intervals	
LOGEC50	1.315 to 1.369
HILLSLOPE	1.925 to 2.723
EC50	20.64 to 23.38
Goodness of Fit	
Degrees of Freedom	10
R^2	0.9872
Absolute Sum of Squares	135.7
Sy.x	3.683
Data	
Number of X values	4
Number of Y replicates	3
Total number of values	12
Number of missing values	0

Figure S16. IC_{50}-Determination of compound NP-013330 (**3**). The absolute IC_{50}-value was determined by nonlinear regression analysis employing GraphPad Prism 3.00 and is given in Table 1.

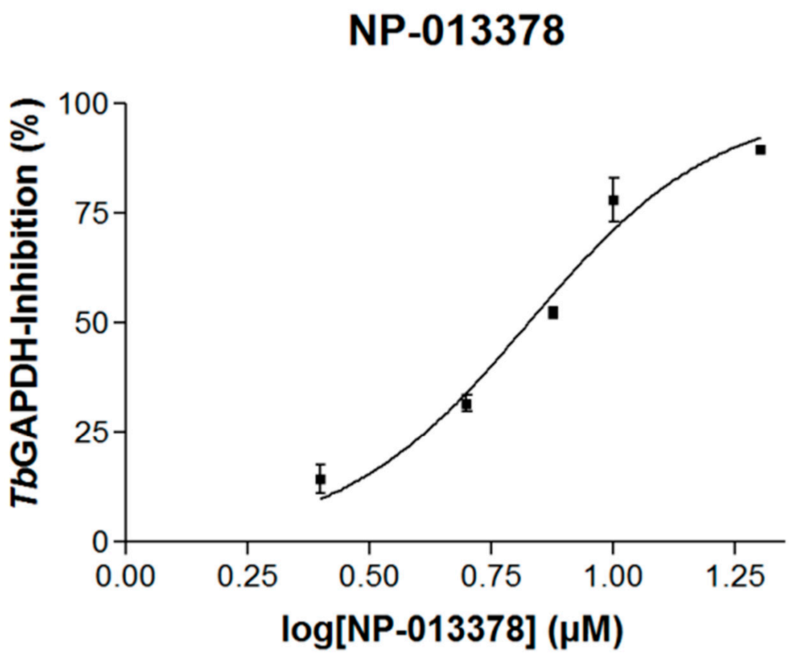

Best-fit values	
BOTTOM (Constant)	0.0
TOP (Constant)	100.0
LOGEC50	0.8249
HILLSLOPE	2.259
EC50	6.683
Std. Error	
LOGEC50	0.01753
HILLSLOPE	0.2323
95% Confidence Intervals	
LOGEC50	0.7871 to 0.8628
HILLSLOPE	1.758 to 2.761
EC50	6.125 to 7.292
Goodness of Fit	
Degrees of Freedom	13
R^2	0.9557
Absolute Sum of Squares	531.4
Sy.x	6.394
Data	
Number of X values	5
Number of Y replicates	3
Total number of values	15
Number of missing values	0

Figure S17. IC_{50}-Determination of compound NP-013378 (**4**). The absolute IC_{50}-value was determined by nonlinear regression analysis employing GraphPad Prism 3.00 and is given in Table 1.

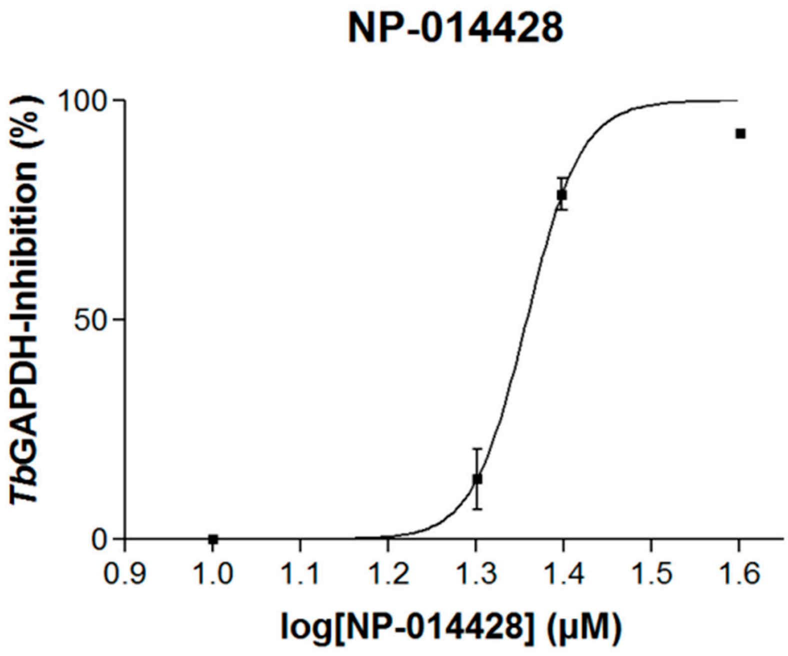

Best-fit values	
BOTTOM (Constant)	0.0
TOP (Constant)	100.0
LOGEC50	1.358
HILLSLOPE	14.08
EC50	22.79
Std. Error	
LOGEC50	0.006392
HILLSLOPE	1.942
95% Confidence Intervals	
LOGEC50	1.344 to 1.372
HILLSLOPE	9.750 to 18.40
EC50	22.06 to 23.55
Goodness of Fit	
Degrees of Freedom	10
R^2	0.9731
Absolute Sum of Squares	527.8
Sy.x	7.265
Data	
Number of X values	4
Number of Y replicates	3
Total number of values	12
Number of missing values	0

Figure S18. IC_{50}-Determination of compound NP-014428 (**5**). The absolute IC_{50}-value was determined by nonlinear regression analysis employing GraphPad Prism 3.00 and is given in Table 1.
